# Editorial: Co-morbidity of COVID 19 and fungal infections

**DOI:** 10.3389/ffunb.2024.1462172

**Published:** 2024-09-16

**Authors:** Ramendra Pati Pandey, Ruby Dhiman, Vivek Mishra, V. Samuel Raj, Chung-Ming Chang

**Affiliations:** ^1^ School of Health Sciences and Technology (SoHST), UPES, Dehradun, Uttarakhand, India; ^2^ Amity Institute of Click-Chemistry Research and Studies (AICCRS), Amity University Uttar Pradesh, Noida, India; ^3^ Centre for Drug Design Discovery and Development (C4D), SRM University, Sonepat, Haryana, India; ^4^ Master & Ph.D. Program in Biotechnology Industry, Chang Gung University, Taoyuan, Taiwan; ^5^ Department of Medical Biotechnology and Laboratory Science, Chang Gung University, Taoyuan, Taiwan

**Keywords:** co-morbidity, COVID-19, fungal infections, clinical management, morbidity and mortality

The World Health Organization (WHO) reports that since the end of 2019, the new severe acute respiratory syndrome coronavirus-2 (SARS-CoV-2) has created a pandemic with 573 million total illnesses and reported cumulative fatalities of 6 million until July 2022. In 2020 (n = 680,894), the total mortality rate was around 3.9%. New variations are continually arising and spreading around the world on a regular basis, despite the development of new treatments and vaccinations. According to a June 2023 update by Hlaing et al., candidemia was a significant co-morbidity among patients who were in severe condition during the COVID-19 pandemic. Initial reports from May 2020 stated that individuals receiving tocilizumab had candidemia.

Later research revealed bloodstream infections in intensive care unit patients, of which candidemia constituted a significant portion (Trieu et al.). Numerous species of *Candida* were shown to be involved, mostly in patients receiving corticosteroids, vasopressor treatment, and mechanical ventilation. These species included *Candida parapsilosis* complex. In an Indian hospital’s intensive care unit, *Candida auris* was responsible for 67% of instances of candidemia, frequently following extended hospital stays. This highlights the crucial role that fungal infections play in the prognosis of COVID-19 patients. In April 2022, Khajotia et al. mentioned that uncontrolled hyperglycemia and the indiscriminate use of corticosteroids are closely associated with the increased prevalence of mucormycosis, which has a 50% death rate in COVID-19 patients (Babamahmoodi et al.; Su et al.). Steroids, while reducing lung inflammation and mitigating cytokine storms in COVID-19, also lower immunity (Tyagi et al., 2022). Rhino-orbital mucormycosis has been notably prevalent in the Indian subcontinent. Reports from institutions and studies indicate concurrent COVID- 19 and mucormycosis infections, with many patients developing diabetic ketoacidosis and requiring intravenous dexamethasone (Sadhu et al., 2021). Despite treatment with liposomal Amphotericin B, outcomes were severe, including death, permanent vision loss, and drastic surgical interventions, highlighting the critical role of fungal co-infections in COVID-19 patients. A summary of fungal infections as a prominent co-morbidity in COVID-19 patients was provided by Kanj et al. in 2023. For candidiasis, first-line treatments are echinocandins (anidulafungin, caspofungin, and micafungin) and azoles (fluconazole or voriconazole), with alternative drugs including lipid formulations of amphotericin B or amphotericin B deoxycholate. Mucormycosis, notably prevalent in the COVID-19 context, is primarily treated with lipid formulations of amphotericin B, while alternatives include amphotericin B deoxycholate, azoles (isavuconazole, posaconazole), and echinocandins in combination with amphotericin B. Aspergillosis management involves first-line drugs like isavuconazole and voriconazole, with alternative treatments including echinocandins (particularly in combination), lipid formulations of amphotericin B (or higher doses of amphotericin B deoxycholate), and other azoles such as posaconazole, itraconazole, and super bioavailability itraconazole (Dutt et al., 2023).

In 2022, Akhtar et al. explained it in great detail. Fungal infections, especially those resulting from the species *Candida*, have been identified as important co-morbidities in individuals with COVID-19. Opportunistic pathogens like *Candida* exacerbate health conditions, especially in immunocompromised individuals. A UK study found 21.4% of respiratory samples from COVID-19 patients tested positive for secondary *Candida* infections. Oral manifestations and xerostomia are common, complicating oral hygiene. Non-*albicans Candida* species, such as *C. auris* and *C. glabrata*, are increasingly reported, often in ICU patients with prolonged stays and invasive devices (Hameed et al., 2021). These infections, often resistant to treatments like amphotericin B and fluconazole, significantly increase mortality rates, highlighting the critical impact of fungal co-infections on COVID-19 outcomes.

A comprehensive strategy is required to effectively prevent fungal co-infections in COVID-19 patients. Improved antifungal therapies are paramount, requiring the development of novel drugs or enhancement of existing ones to combat resistant pathogens like *C. auris* and *C. glabrata*. Concurrently, optimizing the use of corticosteroids demands studies on dosage and duration to minimize immunosuppression and mitigate the risks of mucormycosis and candidemia (Dutt et al., 2023). Early detection strategies, focusing on biomarkers and diagnostic tools, are crucial in intensive care settings to promptly identify fungal infections. Establishing antifungal resistance surveillance programs is essential to monitoring resistance patterns and guiding treatment decisions (Pandey et al., 2021). Preventive strategies in ICUs, including stringent infection control measures and environmental hygiene protocols, are critical to reducing fungal transmission. Exploring immunomodulatory therapies can enhance immune responses against fungal pathogens without compromising COVID-19 treatment efficacy. Developing evidence-based clinical management guidelines tailored to address specific risk factors, such as diabetes and prolonged hospital stays, is imperative (Ruby et al., 2023).

Public health awareness initiatives are needed to educate healthcare providers and the public about the risks and symptoms of fungal infections in COVID-19 patients, advocating for early intervention. Facilitating multidisciplinary collaboration among infectious disease specialists, microbiologists, immunologists, and clinicians is essential to optimizing treatment strategies and outcomes for fungal co-infections in COVID-19. Lastly, research focusing on the pathogenesis of fungal co-infections in COVID-19 patients is crucial. Understanding how SARS-CoV-2 infection predisposes individuals to fungal infections can identify potential therapeutic targets and interventions. Together, these strategies form a comprehensive framework to address the complex intersection of COVID-19 and fungal co-infections, aiming to improve patient outcomes and reduce morbidity and mortality associated with these dual infections.
